# Supplementary material for: Unhealthy Eating Habits and Determinants of Diet Quality in Primary Healthcare Professionals in Poland: A Cross-Sectional Study
Source: Nutrients. 2024 Oct 3;16(19):3367. doi: 10.3390/nu16193367 (PMC11478428; doi:10.3390/nu16193367)
Supplement: Supplementary file 1 [file nutrients-16-03367-s001.zip › nutrients-3218441-supplementary.pdf]

Supplementary Materials:

Table S1. Characteristics of Dietary Quality Score (DQS)

Food	Frequency	Score
Vegetables	>5 servings/week	2 points
	2-5 servings/week	1 point
	<2 servings/week	0 point
Fruit	>3 pieces/day	2 points
	>3 pieces/week and <2 pieces/day	1 point
	<3 pieces/week	0 point
Fish	>200 g/week	2 points
	<200 g/week	1 point
	No intake	0 point
Fat	None	2 points
Fat, spread	Vegetable margarine	1 point
	Butter, blended spread, lard	0 point
Fat, cooking	None/olive oil	2 points
	Vegetable margarine, oil	1 point
	Margarine/butter/blended spread/ lard	0 point
Fat, summarized	6 points, summarized	2 points
	3-5 points, summarized	1 point
	2 points, summarized	0 point

Table S2. Dietary Quality Score categories

Category	Score
Unhealthy dietary habits	0-3 points
Average dietary habits	4-6 points
Healthy dietary habits	7-8 points

Table S3. The odds ratio (OR) of unhealthy dietary habits by individual characteristics (Total).

**Variable**	**Total**	**Unhealthy Dietary Habits**		**Univariable Logistic Regression**		**Multivariable Logistic Regression**	
	N=492 (%)	n=127 (%)	*p*-Value	OR- Odds ratio	95% CI-confidence intervals	OR- Odds ratio	95% CI-confidence intervals
Age (years)							
<40	102 (20.7)	25 (19.7)	0.3964	1.00	Ref.		
40-54	200 (40.7)	58 (45.7)		1.26	(0.73-2.17)		
55+	190 (38.6)	44 (34.6)		0.93	(0.53-1.63)		
Sex							
Female	450 (91.5)	117 (92.1)	0.7564	1.12	(0.54-2.36)		
Male	42 (8.5)	10 (7.9)		1.00	Ref.		
Body mass index BMI							
<25 kg/m^2	242 (49.2)	68 (53.6)	0.1291	1.00	Ref.	1.00	Ref.
≥25–<30 kg/m^2	175 (35.6)	36 (28.3)		0.66	(0.42-1.05) *	0.64	(0.40-1.03)
≥30 kg/m^2	75 (15.2)	23 (18.1)		1.13	(0.64-1.99)	1.08	(0.61-1.92)
Number of chronic diseases							
0	160 (32.5)	37 (29.1)	0.6458	1.00	Ref.		
1	157 (31.9)	46 (36.2)		1.38	(0.83-2.28)		
2	74 (15.1)	19 (15.0)		1.15	(0.61-2.17)		
≥3	101 (20.5)	25 (19.7)		1.09	(0.61-1.96)		
Medical practice							
Private	223 (45.3)	55 (43.3)	0.5958	1.00	Ref.		
Public	269 (54.7)	72 (56.7)		1.12	(0.74-1.68)		
Years of work							
<10	164 (33.3)	37 (29.1)	0.4574	1.00	Ref.		
10-20	145 (29.5)	38 (29.9)		1.22	(0.72-2.05)		
>20	183 (37.2)	52 (41.0)		1.36	(0.84-2.22)		

Number of patient vistis during the routine working week							
≤100	346 (70.3)	98 (77.1)	0.0501	1.59	(0.99-2.55) *	1.58	(0.98-2.55)
>100	146 (29.7)	29 (22.9)		1.00	Ref.	1.00	Ref.
Appropriate training to provide counseling on nutrition, physical activity and weight management							
Yes	415 (84.3)	110 (86.6)	0.4148	1.27	(0.71-2.28)		
No	77 (15.7)	17 (13.4)		1.00	Ref.		
Making measurements of body weight, height, BMI							
Yes	350 (71.1)	90 (70.9)	0.9374	0.98	(0.63-1.53)		
No	142 (28.9)	37 (29.1)		1.00	Ref.		
Tobacco smoking							
Yes	74 (15.0)	25 (19.7)	0.0892	1.58	(0.93-2.69) *	1.54	(0.89-2.66)
No	418 (85.0)	102 (80.3)		1.00	Ref.	1.00	Ref.
E-cigarette use							
Yes	45 (9.1)	9 (7.1)	0.3499	0.69	(0.33-1.49)		
No	447 (90.9)	118 (92.9)		1.00	Ref.		
Alcohol consumption							
Yes	205 (41.7)	50 (39.4)	0.5422	0.88	(0.58-1.33)		
No	287 (58.3)	77 (60.6)		1.00	Ref.		
Physical activity							
Yes	277 (56.3)	66 (52.0)	0.2531	0.79	(0.53-1.18)		
No	215 (43.7)	61 (48.0)		1.00	Ref.		
Number of jobs							
1	378 (76.8)	101 (79.5)	0.4027	1.00	Ref.		
≥2	114 (23.2)	26 (20.5)		0.81	(0.49-1.33)		
Number of meals during the day							

≤2	17 (3.5)	5 (3.9)	0.2746	0.61	(0.17-2.21)	0.58	(0.15-2.16)
3	123 (25.0)	33 (26.0)		0.53	(0.22-1.27)	0.58	(0.24-1.38)
4-5	325 (66.1)	78 (61.4)		0.46	(0.20-1.03) *	0.49	(0.22-1.12)
≥6	27 (5.4)	11 (8.7)		1.00	Ref.	1.00	Ref.

$p < 0.05$*; Ref-reference; Adjusted model, including all statistically significant data.

Table S3. The odds ratio (OR) of unhealthy dietary habits by individual characteristics (GP doctor)- continue.

Variable	GP doctor	Unhealthy Dietary Habits		Univariable Logistic Regression		Multivariable Logistic Regression	
	n=161 (%)	n=28 (%)	*p*-Value	OR- Odds ratio	95% CI- confidence intervals	OR- Odds ratio	95% CI- confidence intervals
Age (years)							
<40	55 (34.2)	10 (35.7)	0.7327	1.00	Ref.		
40-54	62 (38.5)	12 (42.9)		1.08	(0.43-2.74)		
55+	44 (27.3)	6 (21.4)		0.71	(0.24-2.14)		
Sex							
Female	119 (73.9)	18 (64.3)	0.2018	0.57	(0.24-1.36)		
Male	42 (26.1)	10 (35.7)		1.00	Ref.		
Body mass index BMI							
<25 kg/m ^{2}	93 (57.8)	19 (67.9)	0.1025	1.00	Ref.		
≥25–<30 kg/m ^{2}	49 (30.4)	4 (14.3)		0.35	(0.11-1.08) *		
≥30 kg/m ^{2}	19 (11.8)	5 (17.8)		1.39	(0.45-4.34)		
Number of chronic diseases							
0	59 (36.6)	8 (28.6)	0.5750	1.00	Ref.		
1	45 (28.0)	8 (28.6)		1.38	(0.47-4.01)		
2	27 (16.8)	7 (25.0)		2.23	(0.71-6.97)		
≥3	30 (18.6)	5 (17.8)		1.27	(0.38-4.30)		
Medical practice							

Private	104 (64.6)	19 (67.9)	0.6914	1.00	Ref.
Public	57 (35.4)	9 (32.1)		0.84	(0.35-2.00)
Years of work					
<10	46 (28.6)	8 (28.6)	0.8648	1.00	Ref.
10-20	40 (24.8)	8 (28.6)		1.19	(0.40-3.52)
>20	75 (46.6)	12 (42.8)		0.90	(0.34-2.41)
Number of patient vistis during the routine working week					
≤100	59 (36.6)	14 (50.0)	0.1066	1.96	(0.86-4.45)
>100	102 (63.4)	14 (50.0)		1.00	Ref.
Appropriate training to provide counseling on nutrition, physical activity and weight management					
Yes	122 (75.8)	23 (82.1)	0.3870	1.58	(0.56-4.48)
No	39 (24.2)	5 (17.9)		1.00	Ref.
Making measurements of body weight, height, BMI					
Yes	125 (77.6)	23 (82.1)	0.5292	1.40	(0.49-3.98)
No	36 (22.4)	5 (17.9)		1.00	Ref.
Tobacco smoking					
Yes	14 (8.7)	3 (10.7)	0.6766	1.33	(0.35-5.12)
No	147 (91.3)	25 (89.3)		1.00	Ref.
E-cigarette use					
Yes	25 (15.5)	3 (10.7)	0.4391	0.61	(0.17-2.18)
No	136 (84.5)	25 (89.3)		1.00	Ref.
Alcohol consumption					
Yes	68 (42.2)	11 (39.3)	0.7280	0.86	(0.38-1.98)
No	93 (57.8)	17 (60.7)		1.00	Ref.
Physical activity					
Yes	126 (78.3)	19 (67.9)	0.1420	0.51	(0.21-1.26)

No	35 (21.7)	9 (32.1)		1.00	Ref.		
Number of jobs							
1	107 (66.5)	19 (67.9)	0.8632	1.00	Ref.		
≥2	54 (33.5)	9 (32.1)		0.93	(0.39-2.21)		
Number of meals during the day							
≤2	6 (3.7)	-	0.2383	-	-		
3	44 (27.3)	11 (39.3)		1.75	(0.74-4.11)		
4-5	106 (65.8)	17 (60.7)		1.00	Ref.		
≥6	5 (3.2)	-		-	-		

$p < 0.05$*; Ref-reference; Adjusted model, including all statistically significant data.

Table S3. The odds ratio (OR) of unhealthy dietary habits by individual characteristics (Nurse)- continue.

**Variable**	**Nurse**	**Unhealthy Dietary Habits**		**Univariable Logistic Regression**		**Multivariable Logistic Regression**	
	n=331 (%)	n=99 (%)	*p*-Value	OR- Odds ratio	95% CI-confidence intervals	OR- Odds ratio	95% CI-confidence intervals
Age (years)							
<40	47 (14.2)	15 (15.1)	0.3845	1.00	Ref.		
40-54	138 (41.7)	46 (46.5)		1.07	(0.53-2.17)		
55+	146 (44.1)	38 (38.4)		0.75	(0.37-1.54)		
Body mass index BMI							
<25 kg/m ^{2}	149 (45.0)	49 (49.5)	0.3703	1.00	Ref.		
≥25–<30 kg/m ^{2}	126 (38.1)	32 (32.3)		0.69	(0.41-1.18)		
≥30 kg/m ^{2}	56 (16.9)	18 (18.2)		0.97	(0.50-1.86)		
Number of chronic diseases							

0	101 (30.5)	29 (29.3)	0.6906	1.00	Ref.
1	112 (33.8)	38 (38.4)		1.28	(0.71-2.28)
2	47 (14.2)	12 (12.1)		0.85	(0.39-1.87)
≥3	71 (21.5)	20 (20.2)		0.97	(0.50-1.91)
Medical practice					
Private	119 (36.0)	36 (36.4)	0.9187	1.00	Ref.
Public	212 (64.0)	63 (63.6)		0.97	(0.60-1.59)
Years of work					
<10	118 (35.7)	29 (29.3)	0.1160	1.00	Ref.
10-20	105 (31.7)	30 (30.3)		1.23	(0.68-2.23)
>20	108 (32.6)	40 (40.4)		1.81	(1.02-3.20) *
Number of patient vistis during the routine working week					
≤100	287 (86.7)	84 (84.8)	0.5153	0.80	(0.41-1.57)
>100	44 (13.3)	15 (15.2)		1.00	Ref.
Appropriate training to provide counseling on nutrition, physical activity and weight management					
Yes	293 (88.5)	87 (87.9)	0.8111	0.92	(0.44-1.90)
No	38 (11.5)	12 (12.1)		1.00	Ref.
Making measurements of body weight, height, BMI					
Yes	225 (68.0)	67 (67.7)	0.9393	0.98	(0.59-1.62)
No	106 (32.0)	32 (32.3)		1.00	Ref.
Tobacco smoking					
Yes	60 (18.1)	22 (22.2)	0.2064	1.29	(0.74-2.24)
No	271 (81.9)	77 (77.8)		1.00	Ref.
E-cigarette use					
Yes	20 (6.0)	6 (6.1)	0.9927	1.01	(0.37-2.70)
No	311 (94.0)	93 (93.9)		1.00	Ref.

Alcohol consumption					
Yes	137 (41.4)	39 (39.4)	0.6301	0.89	(0.55-1.44)
No	194 (58.6)	60 (60.6)		1.00	Ref.
Physical activity					
Yes	151 (45.6)	47 (47.5)	0.6580	1.11	(0.69-1.78)
No	180 (54.4)	52 (52.5)		1.00	Ref.
Number of jobs					
1	271 (81.9)	82 (82.8)	0.7683	1.00	Ref.
≥2	60 (18.1)	17 (17.2)		0.91	(0.49-1.69)
Number of meals during the day					
≤2	11 (3.3)	5 (5.1)	0.1066	1.00	Ref.
3	79 (23.9)	22 (22.2)		0.46	(0.13-1.67)
4-5	219 (66.2)	61 (61.6)		0.46	(0.14-1.57)
≥6	22 (6.6)	11 (11.1)		1.2	(0.28-5.12)

p < 0.05*; Ref-reference; Adjusted model, including all statistically significant data.
